# Modeling Magnetic Transition Temperature of Rare-Earth Transition Metal-Based Double Perovskite Ceramics for Cryogenic Refrigeration Applications Using Intelligent Computational Methods

**DOI:** 10.3390/ma18194594

**Published:** 2025-10-03

**Authors:** Sami M. Ibn Shamsah

**Affiliations:** Department of Mechanical Engineering, College of Engineering, University of Hafr Al Batin, P.O. Box 1803, Hafr Al Batin 31991, Saudi Arabia; sibnshamsah@uhb.edu.sa

**Keywords:** E_2_TMO_6_ ceramics, extreme learning machine, double oxide perovskite, genetic algorithm, magnetic transition temperature, support vector regression and magnetic field

## Abstract

Rare-earth transition metal-based double perovskite ceramics E_2_TMO_6_ (where E = rare-earth metals, T = transition metals, and M = metal) have received impressive attention lately for cryogenic applications as a result of their intrinsic physical features such as multiferroicity, dielectric features, and adjustable magnetic transition temperature. However, determination and enhancement of magnetic transition temperature of E_2_TMO_6_ ceramic are subject to experimental procedures and processes with a significant degree of difficulties and cumbersomeness. This work proposes an extreme learning machine (ELM)-based intelligent method of determining magnetic transition temperature of E_2_TMO_6_ ceramics with activation function sigmoid (SM) and sine (SE) at varying magnetic field. The outcomes of the SE-ELM and SM-ELM models were compared with genetically optimized support vector regression (GEN-SVR) predictive models using RMSE, CC, and MAE metrics. Using the testing samples of E_2_TMO_6_ ceramics, SE-ELM predictive model outperforms GEN-SVR with a superiority of 6.3% (using RMSE metric) and 15.7% (using MAE metric). The SE-ELM predictive model further outperforms the SM-ELM model, with an improvement of 5.3%, using CC computed with training ceramic samples. The simplicity of the employed descriptors, coupled with the outstanding performance of the developed predictive models, would potentially strengthen E_2_TMO_6_ ceramics exploration for low-temperature cryogenic applications and circumvent energy challenges in different sectors.

## 1. Introduction

Magnetocaloric effect–based magnetic materials offer energy-efficient and next-generation systems of refrigeration with significant potential to revolutionize cooling industries and address global energy challenges [1,2,3]. This system of refrigeration is connected with the intrinsic entropy within magnetic materials [4]. Adiabatic conditional inclusion of magnetic fields within magnetic materials orients the magnetic moment domicile in the material and subsequently results in reduction in magnetic entropy [5,6]. Consequently, the system’s effort to keep the total entropy constant leads to an increase in temperature. Removal of magnetic field results in an increase in magnetic entropy with an ultimate decrease in temperature. Magnetic cooling systems utilize the concept of temperature fluctuation due to application/removal of a magnetic field [7,8,9]. This cooling system is thermodynamically efficient and promotes environmental friendliness since it circumvents utilization of ozone-depleting refrigerants [10]. Beyond cooling applications, magnetocaloric effect-based magnetic materials find applications in other cryogenic domains such as magnetic sensors operating at cryogenic temperatures, spin valves, and magnetic memory devices for data storage, among others [11]. The magnetic parameters that significantly influence the candidature of any magnetic materials for cryogenic applications include the magnetocaloric effect, magnetic cooling power, and magnetic transition temperature. Magnetic transition temperature plays a significant role in regulating transition between ferromagnetism to paramagnetism. The absence of magnetocaloric compounds with desired features for cryogenic applications hinders commercialization of the technology [12]. Therefore, discovery of new materials capable of displaying a large magnetocaloric effect and magnetic transition temperature is of significant interest for cryogenic applications. Rare-earth-based compounds have received overwhelming attention lately as novel magnetocaloric materials due to their huge magnetic moments, excellent ferromagnetic interaction, and impressive magnetic properties associated with their localized f-orbital electrons [13,14,15,16]. Super-exchange interactions in rare-earth-based compounds create an avenue for a sharp paramagnetic to ferromagnetic transition. Recent studies utilized computational intelligence approaches to explore the magnetocaloric effect [17] and magnetic cooling power [18] of these compounds for cryogenic applications, while magnetic transition temperature remains unexplored. Magnetic transition temperature is the temperature at which phase transition occurs within the magnetic materials from ferromagnetic to paramagnetic state. This temperature is a critical parameter, as it determines potential applications of magnetocaloric materials for cryogenic applications. Several physical complications are associated with magnetic behavior of double perovskite compounds modeled in this work. In the temperature range near the phase transition, multiple magnetic phases co-exist, and three different magnetic phase transitions were observed and reported in R_2_NiMnO_6_ compound [19]. The magnetic phase transitions were attributed to rare-earth ordering, 3d-4d exchange, and 3d metal ordering. Similarly, a paramagnetic state of double perovskite-based compounds has been reported to have a magnetic phase known as Griffiths phase with direct influence on magnetic properties of double perovskite compounds [20]. In order to simplify these associated magnetic phases, only one phase is presently simulated in this work. This work develops computational intelligent methods of determining magnetic transition temperature of E_2_TMO_6_ rare-earth transition metal-based double perovskite ceramics.

Over the years, conventional magnetocaloric effect materials have seen extensive investigation. These magnetic materials, including spinel ferrites, europium titanate, manganite, and gadolinium alloys, have demonstrated strong magnetocaloric properties that are important parameters for assessing the cryogenic ability of a material [16,21,22,23,24,25,26]. However, large-scale fabrication and wide adoption have been constrained due to chemical stability, cost-effectiveness, and cryogenic operational temperature, among others. Recently, rare-earth-based oxides have been explored as a new class of material to mitigate these drawbacks. Specifically, double perovskite oxides with the general formula E_2_TMO_6_ (where E is a rare-earth metal, T is a transition metal, and M is another metal) present exciting research interest [27]. Besides their elevated MCE and RCP, these ceramics displayed intriguing physical properties that hold potential for cryogenic applications. However, magnetic transition temperature of these ceramics has not been investigated in the literature using computational approaches. These materials are derived from the fundamental ABO_3_ perovskite structure [28], where a large cation (A-site) is surrounded by twelve oxygen ions, and a smaller cation (B-site) is at the center of six oxygen octahedra [19,29]. In the double perovskite structure, the B-site is occupied by two different cations, T and M, which allows for a vast array of compositional permutations. This structural flexibility is crucial, as altering the ionic radii and electronic configurations of the constituent E, T, and M metals can induce fascinating magnetic behaviors, including spin-phonon coupling and field-induced metamagnetic transitions, which directly influence their magnetic transition temperature [30,31]. The advantage of ionic radii descriptors as compared to other descriptors such as magnetic moment of ions, tolerance factor, and bond angle Mn-O-Mn (which are known to influence magnetic properties of double perovskite compounds) is the simplicity, which promotes pre-laboratory simulation without any experimental procedures with sophisticated equipment. While experimental studies have confirmed their potential, a systematic method for predicting and optimizing the magnetic transition temperature across a vast range of possible alloys remains a point of research interest. This work aims to contribute to this gap by employing intelligent computational models (support vector regression optimized by genetic algorithm and extreme learning machine) to investigate and predict magnetic transition temperature of these perovskites and therefore develop a framework for the rational design of new E_2_TMO_6_ with high and tunable magnetic transition temperature.

Support vector regression (SVR) is a class of supervised learning-based intelligent algorithms for addressing regression problems [32,33]. The algorithm employs structural risk principle of minimization for controlling and minimizing training error bounds. The mathematical framework of SVR employs Lagrange multipliers in addressing convex optimization problems and transforms data samples to a space of high dimension where data samples are linearly analyzed [34]. This transformation mechanism allows SVR to conveniently address nonlinear and challenging science as well as engineering problems [35,36,37]. To further control the precision and accuracy of the prediction outcomes of SVR-based predictive models, parameters such as the penalty factor, the parameter associated with nonlinear mapping function called kernel parameter, and epsilon are adjusted in a combinatory manner. Genetic evolutionary algorithm has been employed for this parametric optimization. Genetic algorithm is a population-based search heuristic algorithm for locating optimal solutions in a complex optimization system [38,39]. The algorithm is based on the process of natural selection, inspired by the popular theory of natural evolution. The choice of genetic algorithm in this work is due to the robustness of the algorithm coupled with flexibility as well as absence of premature convergence [40]. The performance of genetically optimized support vector regression (GEN-SVR)-based predictive models was assessed using performance metrics such as the mean absolute error (MAE), correlation coefficient (CC), and root mean square error (RMSR) for training as well as testing E_2_TMO_6_ rare-earth transition metal-based double perovskite ceramic samples for magnetic transition temperature prediction.

Extreme learning machine (ELM) is a modified and upgraded version of feedforward neural network characterized by the presence of a single hidden layer with reduced computational complexity [41,42]. It employs empirical principle of minimizing training error range and is known for characteristic fast as well as efficient learning. As compared with the conventional backpropagation learning system, ELM enjoys rapid training speed with simple and excellent generalization [43]. The algorithm utilizes random weights for its input weights, while only output weights are subjected into training processes. Implementation of extreme learning machine algorithm involves the choice of activation function with the number of neurons, initialization of random weights, computation of the output of the hidden layer, computation of output weights, and prediction of the desired material properties [44]. The uniqueness of this algorithm strengthens its applications in science and engineering domains. These unique features are harnessed in this work in determining the magnetic transition temperature of E_2_TMO_6_ rare-earth transition metal-based double perovskite ceramics.

The results of modeling indicate that sine function-based ELM (SE-ELM) predictive model outperforms the sigmoid activation function-based ELM (SM-ELM) and genetically optimized SVR (GEN-SVR) predictive models using different performance metrics such as correlation coefficient (CC), mean absolute error (MAE), and root mean square error (RMSE). For the testing samples of E_2_TMO_6_ ceramics, SE-ELM outperforms the SM-ELM predictive model with improvements of 26.579% (using CC metric), 53.2679% (using RMSE metric), and 51.5744% (using MAE metric). Similarly, GEN-SVR performs better than the SM-ELM model, with an improvement of 5.3005%, using CC computed with training samples of rare-earth transition metal-based double perovskite ceramics. Using testing samples of rare-earth transition metal-based double perovskite ceramics, the GEN-SVR predictive model performs better than the SM-ELM model, with an improvement of 23.3501% (using CC metric), 50.1124% (using the RMSE metric), and 42.5786% (using MAE metric).

The remaining part of the manuscript is organized as follows: section two describes the mathematical framework of the intelligent algorithm, while section three discusses the computational strategies coupled with a description of E_2_TMO_6_ rare-earth transition metal-based double perovskite ceramic samples. The fourth section presents the outcomes of the predictive models and compares the predictions with the measured magnetic transition temperatures. The last section summarizes the findings of the research work.

## 2. Mathematical Formulation of the Intelligent Algorithms Implemented

The section contains the presentation of mathematical foundation of intelligent algorithms. The algorithms include support vector regression, extreme learning machine, and genetic algorithm.

### 2.1. Support Vector Regression Mathematical Background

A potent machine learning method for forecasting continuous target variables is support vector regression (SVR) [45,46]. Support vector machines, initially developed for classification problems, form the foundation of SVR. In the context of regression, SVR looks for a function that, while keeping a healthy margin of error, best approximates the connection between the target variable and input descriptors [47]. SVR’s main concept is that the defined descriptors are mapped into a new space with higher dimensions using a nonlinear kernel function. This enables the algorithm to identify intricate associations in the data that conventional linear regression models would overlook. It seeks to create a hyperplane in the feature space that, within a tolerance margin, minimizes the disparity between the measured and predicted magnetic transition temperature.

Modeling nonlinear relationships without explicitly defining the type of nonlinearity is a major benefit of SVR over traditional regression models. SVR can function in a feature space without transformation computation in an explicit manner through kernel utilization, such as the Gaussian and polynomial kernels, which makes it computationally efficient [48,49]. The mathematical foundation of SVR is hereby presented. In order to efficiently model and predict the underlying patterns and dependencies in magnetic transition temperature and descriptors (which include the applied magnetic field, ionic radii of rare-earth metal, transition metal, and other metal for E_2_TMO_6_ rare-earth transition metal-based double perovskite ceramics, SVR uses the function given in Equation (1):

(1) $$\gamma \left(d\right)=\langle \alpha ,\;d\rangle +\varphi .$$

Here, *γ* denotes the SVR model’s projected output (predicted magnetic transition temperature, and d represents the descriptors, which include the applied magnetic field, ionic radii of rare-earth metal, transition metal, and other metal. The essential elements of the model, the weight vector (*α*), and bias (*φ*) coefficients are calculated by minimizing the risk function given in Equation (2) [50].

(2) $$r\left(\gamma \right)=\frac{s}{t}\sum_{n=1}^{m}L\left(\gamma \left(d\right)-\;\;\gamma_{n}^{*}\right)+\;\frac{∥∁\;{∥}^{2}}{2}$$

Deviations above the epsilon threshold are penalized by the regularization factor, represented by the symbol s. The Euclidean norm is represented by $∥\;∁\;{∥}^{2}$ in Equation (2), while the error threshold is defined by L, as Equation (3) illustrates.

(3) $$L\left(\gamma \left(d\right)-\gamma^{*}\;\right)=\;\left\{\begin{array}{c} ∥\gamma \left(d\right)-\;\;\gamma^{*}∥-\partial \;|\gamma \left(d\right)-\;\;\gamma^{*}|\geq \partial \\ \;0\;\gamma \left(d\right)-\;\;\gamma^{*}<\partial \end{array}\right.$$

Slack variables δ and δ* must be included, particularly when there is a chance that the error threshold will be surpassed. These variables partially regulate the gap between the defined bounds and the observed values. To address the dual problem, the optimization process was carried out using the procedures outlined in Equation (4) and is constrained by the conditions stated in Equation (5):

(4) $$Minimize:\;\frac{1}{2}\sum_{n}^{m}\sum_{l}^{m}\left(\sigma_{n}-\sigma_{n}^{*}\right)\left(\sigma_{l}-\sigma_{l}^{*}\right)\lambda \left(d_{n},\;d_{l}\right)+\sum_{n=1}^{m}\gamma_{n}^{*}\left(\sigma_{n}-\sigma_{n}^{*}\right)-\tau \sum_{n=1}^{m}\left(\sigma_{n}+\sigma_{n}^{*}\right),$$

(5) $$\sum_{n=1}^{m}\left(\sigma_{n}+\sigma_{n}^{*}\right)=0,\sigma_{n},\sigma_{n}^{*}\;\epsilon \left[0,s\right],$$

where the Lagrange multipliers utilized to solve the optimization are denoted by $\sigma_{n}$ and $\sigma_{n}^{*}$. The data points with coefficients which are non-zero are referred to as support vectors. Equation (6) shows the expected values produced by the algorithm using optimization procedures.

(6) $$\gamma \left(d\right)=\sum_{n=1}^{m}\left(\sigma_{n}-\sigma_{n}^{*}\right)\langle d_{n},\;d\rangle +\rho$$

The kernel function is essential for transforming the initial nonlinear regression into a linear one in the new space. The mapping function described in Equation (7) captures this transition, and Equation (8) expresses the nonlinear regression model’s final form in the SVR framework [51].

(7) $$\gamma \left(d_{n},d_{l}\right)=\;\vartheta \left(d_{n}\right)\vartheta \left(d_{l}\right)\;$$

(8) $$\gamma \left(d\right)=\sum_{n=1}^{m}\left(\sigma_{n}-\sigma_{n}^{*}\right)\mu \left(d_{n},d_{l}\right)+\rho$$

### 2.2. Genetic Algorithm Theory and Description

**Figure 1:**
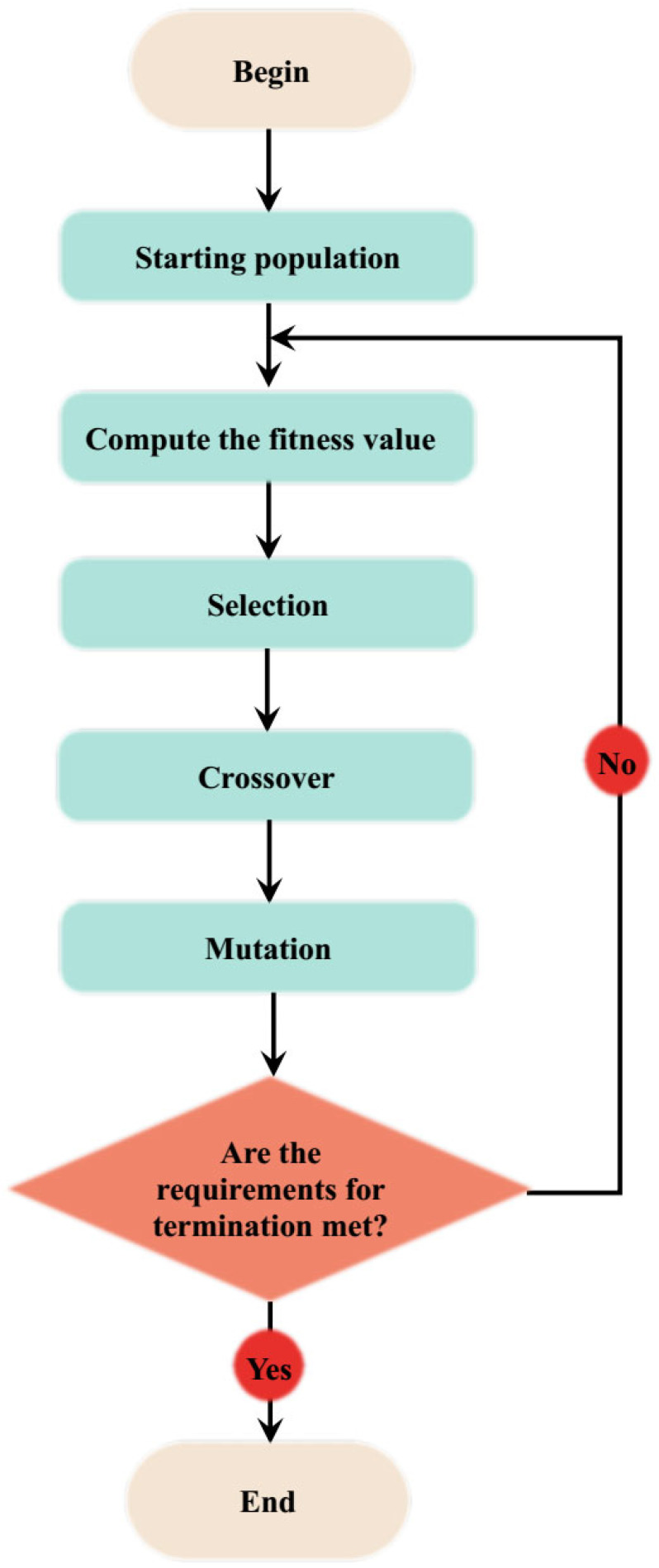
Computational flowchart of the genetic algorithm (GA).

Genetic algorithm (GA) is an evolutionary optimization method-based on the fundamental principle of genetics and natural selection [52]. The algorithm addresses the optimization problem through systemic mimicking of evolution theory with key components such as population initiation, fitness function definition, parent selection, crossover, and mutation. In population initialization, probable solutions are initiated and represented as vectors or strings [53,54]. The quality of the individual solutions is subsequently assessed through fitness function computation. The selection procedures follow, in which most fit solutions are selected for reproduction. In the crossover stage, new offspring are created through parent combination, while the mutation stage further alters the solutions through randomization of genetic constituents [55]. Selection, crossover, and mutation procedures are probabilistic with assigned probability values. Figure 1 presents the flowchart of genetic algorithms.

### 2.3. Extreme Learning Machine Theory and Description

Extreme learning machine (ELM) is classified as a neural network (feed-forward) with a single hidden layer designed to test and train neural networks without using back-propagation to update their weights [42,56,57]. This method is suggested to shorten a model’s training period, which is caused by repeated parameter adjusting procedures. Therefore, ELM can update the weights without performing a back-propagation phase [58]. Let p, q, and z, respectively, stand for the quantity of input features (which include the applied magnetic field, ionic radii of rare-earth metal, transition metal, and other metal), ELM’s outputs (magnetic transition temperature of E_2_TMO_6_ rare-earth transition metal-based double perovskite ceramics), and hidden nodes. Let ($x_{i}$, $d_{i}$) with $x_{i}$ = [$x_{i1}$, $x_{i2}$, …, $x_{ip}$] ∈ $R^{p}$ and $d_{i}$ = [$d_{i1}$, $d_{i2}$, …, $d_{iq}$] ∈ $R^{q}$ be the training sample for *i* = 1, 2, …, P with P is the number of E_2_TMO_6_ ceramics. The predicted magnetic transition temperature ($y_{i}$ ∈ $R^{q}$) for the input descriptors is represented in Equation (9):

(9) $$y_{i}=\;\sum_{j=1}^{z}{\bar{\alpha }}_{j}h_{j}{(X}_{i}),$$

where vector ${\bar{\alpha }}_{j}$ = [$\alpha_{j1}$, $\alpha_{j2}$, …, $\alpha_{jq}$] ∈ $R^{q}$ represents the weight vector joining the j output and hidden nodes, and $h_{j}{(x}_{i})$ depicts the output of hidden node *j*, mathematically represented as $h_{j}{(x}_{i})$) = **δ**($\varphi_{j}$, $b_{j}$, $x_{i}$), where $\varphi_{j}$∈ $R^{p}$ is the weight vector, $b_{j}$ represents bias, and **δ** is the actuation function [59].

The row vector that entails the hidden layer prediction after $K_{i}$ input vector is represented as G($K_{i}$) = [$g_{1}{(K}_{i})g_{2}{(K}_{i})\ldots ,g_{z}{(K}_{i})$] with matrix form representation shown in Equation (10).

(10) $$G\;=\;\left[\begin{array}{c} {g(k}_{1})\\ {g(k}_{2})\\ \begin{array}{c} \vdots \\ {g(k}_{P}) \end{array} \end{array}\right]\;=\;\left[\begin{array}{ccc} g_{1}{(k}_{1}) & g_{2}{(k}_{1}) & \begin{array}{cc} \ldots & g_{z}{(k}_{1}) \end{array}\\ g_{1}{(k}_{2}) & g_{2}{(k}_{2}) & \begin{array}{cc} \ldots & g_{z}{(k}_{2}) \end{array}\\ \begin{array}{c} \vdots \\ g_{1}{(k}_{P}) \end{array} & \begin{array}{c} \vdots \\ g_{2}{(k}_{P}) \end{array} & \begin{array}{cc} \ldots & \begin{array}{c} \vdots \\ g_{z}{(k}_{P}) \end{array} \end{array} \end{array}\right]$$

The predicted magnetic transition temperature is shown in Equation (11):

(11) y = Gα,

where y = [$y_{1},$ $y_{2}$, …,$y_{P}$] ^T^ ∈ $R^{P\times q}$. The algorithm aims to determine α, which is the solution objective for the optimization problem shown in Equation (12) [60]:

(12) $$\min_{\alpha }{\left\|G\alpha -D\right\|}_{F},$$

where **D** = [**d_1_,** **d_2_,** **…,** **d_P_**]^T^ ∈ R^P×q^ is the matrix representing the predicted magnetic transition temperature and $\left\|\cdot \right\|$_F_ is the Frobenius norm. The solution of the given problem is depicted by Equation (13):

(13) $$\hat{\alpha }\;=\;G^{\tau }D,$$

where **G**^τ^ stands for the Moore–Penrose inverse of **G** or the pseudo-inverse.

## 3. Computational Methodology and Approaches

This section contains the acquisition of E_2_TMO_6_ rare-earth transition metal-based double perovskite ceramic samples, the computational hybridization of genetic algorithms with support vector regression, and the computational details of ELM-based algorithms.

### 3.1. E_2_TMO_6_ Rare-Earth Transition Metal-Based Double Perovskite Ceramic Samples Acquisition and Description

E_2_TMO_6_ rare-earth transition metal-based double perovskite ceramic data samples employed for magnetic transition temperature prediction were extracted from the literature [19,29,61,62,63,64,65,66,67]. The data samples consist of twenty-seven magnetocaloric compounds of E_2_TMO_6,_ rare-earth transition-metal-based double perovskite ceramics. The descriptors include the applied magnetic field, ionic radii of the rare-earth metal, transition metal, and other incorporated metals. Statistical analysis was conducted on the data sample, and the reports of the analysis are contained in Table 1.

The computed statistical parameters include the mean values, which entail the overall content of E_2_TMO_6_ rare-earth transition metal-based double perovskite ceramic data samples, maximum as well as the minimum values through which data sample ranges are determined. The analysis also contains the standard deviation, which presents the consistency in the employed data samples, as the data were experimentally measured from different experimental conditions. The computed coefficients of correlation explain the nature of the relationship existing between each of the descriptors and the measured magnetic transition temperature. The applied magnetic field, ionic radii of rare-earth metals, and those of transition metals are negatively correlated with the measured magnetic transition temperature, while the ionic radii of the incorporated metals show positive correlation. The coefficients of correlation of all the descriptors are relatively small compared to the incorporated metal descriptor. These observations indicate that ordinary linear models cannot effectively capture the existing relationship between the descriptors and magnetic transition temperature. Hence, choosing nonlinear modeling tools, such as the hybrid SVR and ELM employed, becomes necessary.

### 3.2. Computational Development of Hybrid Genetic Algorithm and Support Vector Regression

E_2_TMO_6_ rare-earth transition metal-based double perovskite ceramics (utilized in developing predictive models through which magnetic transition temperatures are determined and predicted) were randomized and partitioned into training and testing with a 4:1 division ratio. The entire computation was conducted using the computing environment of MATLAB (R2024B). The randomization procedures strengthen even partitioning of the data samples and prevent the likelihood of data disparity in which the model acquires patterns from a part of data samples that cannot be generalized for future prediction. Hybridization of genetic algorithm with support vector regression (GEN-SVR) maximizes regression task performance by combining the advantages of SVR and genetic algorithm. Through this integration, important SVR model parameters like the penalty factor, error epsilon, and kernel parameters are adjusted by utilizing genetic algorithm optimization capabilities. GEN-SVR predictive model has been developed using the following computational procedures:

**Step 1**: *Data partitioning and randomization*: The samples were randomized and partitioned. The process of data partitioning involves splitting the dataset into sets for testing and training; 20% of E_2_TMO_6_ rare-earth transition metal-based double perovskite ceramic data samples were employed for model validation, while 80% of the E_2_TMO_6_ rare-earth transition metal-based double perovskite ceramic samples were utilized for pattern acquisition.

**Step 2**: *Solution space initialization*: The search space for the SVR parameters was initiated using a genetic algorithm. A set of hyperparameters to be optimized is represented by each chromosome. The search space has ranges with upper and lower bounds for the kernel parameter, epsilon, and penalty factor. The upper and lower limits of the penalty factor were defined as 10,000 and 10, respectively, while those of epsilon were set at 1.0 and 0.1. For the Gaussian kernel parameter, upper and lower solution spaces were set at 1.0 and 0.1, respectively.

**Step 3**: *Fitness evaluation*: The fitness of each of the chromosomes within the solution space was computed through root mean square error determination. The procedures for fitness computation involve the following: (i) selecting nonlinear mapping functions from the pool, which includes Gaussian, polynomial, sigmoid, and other functions; (ii) training E_2_TMO_6_ rare-earth transition metal-based double perovskite ceramic samples, together with the chromosome (whose fitness is to be determined), by combining them with the selected mapping function in (i) for magnetic transition temperature determination; (iii) combining the support vectors generated during (ii) with the testing E_2_TMO_6_ rare-earth transition metal-based double perovskite ceramic samples to determine the magnetic transition temperature; and (iv) evaluating the performance/fitness of each chromosome using root mean square error (RMSE) between the measured and predicted magnetic transition temperature. The chromosome with the lowest RMSE fits within the solution space.

**Step 4**: *Subsequent generation through genetic operational principles*: Selection probability of 0.8 was implemented for selecting parents which produce offspring for the subsequent generations. This ensures that most fit chromosomes within the population migrate to the next generation.

**Step 5**: *Crossover implementation:* To produce new offspring with better features, crossover procedures unite segments of specific chromosomes. A crossover probability of 0.9 was employed in order to promote variety and effectively explore the solution space.

**Step 6**: *Mutation operation:* In order to preserve diversity without impeding the convergence process, mutation causes random changes to the chromosomes. Mutation probability was set at 0.005.

**Figure 2:**
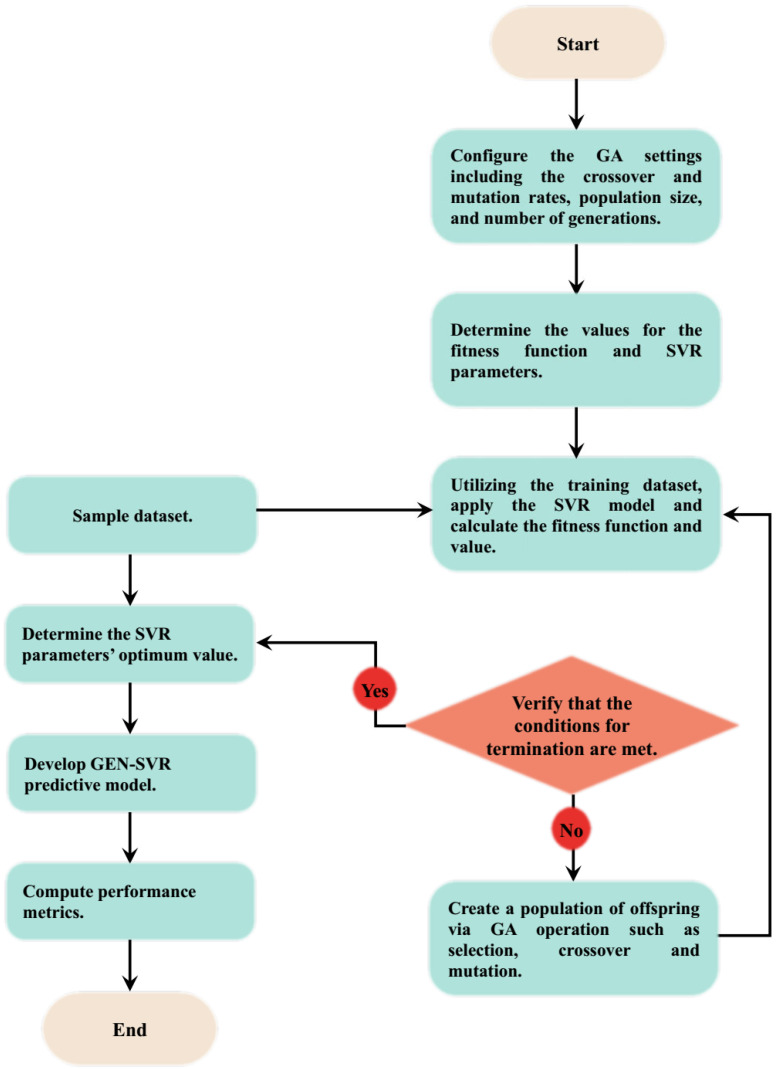
Computational description of GEN-SVR predictive model for magnetic transition temperature determination.

**Step 7**: *Stopping conditions:* The aforementioned six steps were continuously repeated until one of the halting criteria was satisfied. The conditions include reaching a predetermined maximum number of iterations, maintaining the same value of RMSE for 50 consecutive iterations, and obtaining an RMSE value of zero. The computational details are pictorially represented in Figure 2.

### 3.3. Computational Development of ELM-Based Model for Magnetic Transition Temperature Prediction in E_2_TMO_6_ System of Ceramics

ELM-based predictive models were developed for predicting magnetic transition temperature of testing E_2_TMO_6_ rare-earth transition metal-based double perovskite ceramics. The data samples were subjected to randomization processes and then divided into training and testing with a 4:1 division ratio. The computational procedures are outlined as follows:

**Step A**: *Initialization of ELM parameters and configuration of Mersenne Twister generator:* ELM parameters that control the precision of the predictive model were initiated. The parameters include the neuron number and the activation function. Neuron number was optimized using the grid search approach for each selected activation function. Although population-based optimization methods are very efficient for multiple-parameter optimization. Utilization of an evolutionary algorithm for neuron number optimization amounts to a waste of computational resources since only a parameter is to be optimized. The Mersenne Twister generator was initiated and set at a value that was maintained throughout the modeling and simulation stages. Input weights and biases were generated for subsequent use. This process strengthens reproducibility of the model weights.

**Step B**: *Neuron number and activation function selection:* The solution space of the neuron number was defined between 1 and 100 for each activation function. The explored activation functions include the radial basis, sine, sigmoid, transig, and triangle basis functions.

**Step C**: *Weights acquisition for future prediction*: Training samples of E_2_TMO_6_ rare-earth transition metal-based double perovskite ceramics were combined with the outcomes of **Steps A** and **B** to compute output weights using the Moore–Penrose generalized inverse matrix.

**Step D**: *Assessment of the generalization and estimation capacity of the developed model:* In order to evaluate the developed ELM-based predictive models for future generalization, testing ceramics, biases (outcomes of **Step A**), output weights (outcomes of **Step C**), and randomly generated input weights from **Step A** were all employed. The measurement of generalization capacity was achieved by calculating root mean square error (RMSE) involving the extreme learning machine algorithm’s predictions and the measured magnetic transition temperature. Other performance metrics, such as mean absolute error (MAE) and correlation coefficient (CC), were also computed for the testing and training E_2_TMO_6_ rare-earth transition metal-based double perovskite ceramic samples.

**Figure 3:**
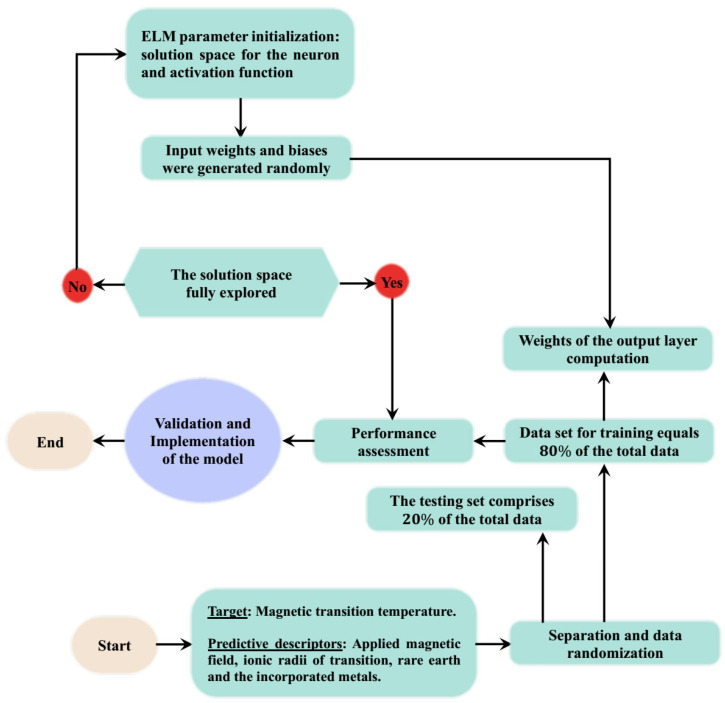
Computational description of the developed SE-ELM and SM-ELM predictive models.

**Step E**: **Steps A** to **D** were repeated for using different activation, while the values of the performance metrics were computed and recorded. Activation functions that show better performance include the sine (SE) and sigmoid (SM) functions which give rise to the SE-ELM and SM-ELM predictive models. Figure 3 presents the computational step-by-step procedures of the developed SE-ELM and SM-ELM predictive models.

## 4. Results and Discussion

In this section, the predictive capacities of the developed SM-ELM, SE-ELM, and GEN-SVR models in determining the magnetic transition temperature of E_2_TMO_6_ ceramics are presented. GA convergence at different chromosome sizes is discussed. The future estimated strength of the predictive models is also presented using different metrics.

### 4.1. Convergence of GEN-SVR Parameters at Different Chromosome Sizes

**Figure 4:**
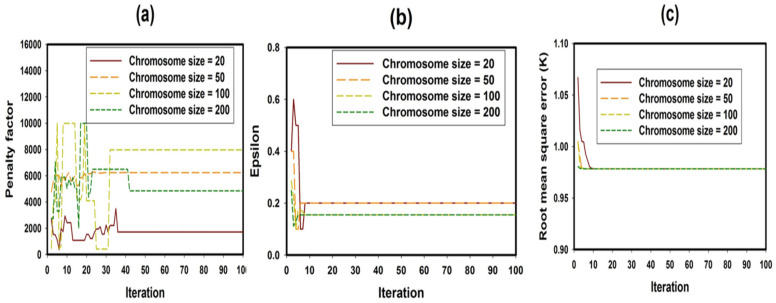
Parameter convergence in GEN-SVR model for (**a**) penalty factor, (**b**) epsilon threshold and (**c**) model root mean square error.

The exploitation and exploration ability of chromosomes within the genetic algorithm operational principle in optimizing the precision tuning parameters in support vector regression algorithm are shown in Figure 4 over a hundred iterations. Genetic algorithm parameters that influence the model accuracy include the population size, crossover rate, and mutation rate, among others. Larger population size strengthens diversity and enhances the exploration of search space, while small population size may result in premature convergence. Crossover rate helps combine genetic materials of two different parents, which results in offspring production. A high crossover rate enhances the mixing of traits and the exploration of new solutions. Low mutation rate promotes stagnation. Optimum choice of these parameters contributes significantly to the model accuracy. Figure 4a and Figure 4b, respectively, present the convergence of the penalty factor and epsilon, while Figure 4c shows the convergence of the fitness function computed using RMSE between the measured magnetic transition temperature and the estimated values for rare-earth transition metal-based double perovskite ceramics. The genetic algorithm avoids premature convergence through a larger population size, which ranges between 20 and 200 in this case. Crossover and mutation rates were also carefully maintained at probabilities of 0.9 and 0.05, respectively, purposely to avoid potential premature convergence.

For the penalty factor convergence presented in Figure 4a, stable convergence was attained after fortieth iteration for all the investigated chromosome sizes. Penalty factor, also known as regularization factor, penalizes data points outside the precision zone. The parameter controls and balances the tradeoff between model complexity and precision. Epsilon convergence shown in Figure 4b describes the user-defined error zone during pattern acquisition. For every investigated chromosome size, stable convergence was attained after tenth iteration. The fitness function, which measures the strength of each individual chromosome, is shown in Figure 4c at different sizes of chromosomes. The convergence of fitness function (determined through root mean square error computation) does not significantly depend on the size of the chromosome and iteration number (since stable convergence appears just before tenth iteration for all the investigated chromosome sizes). Table 2 contains the optimum (global) values for each of the precision tuning parameters in support vector regression algorithm.

### 4.2. Generalization Strength Comparison Between Developed Models Using Different Metrics

**Figure 5:**
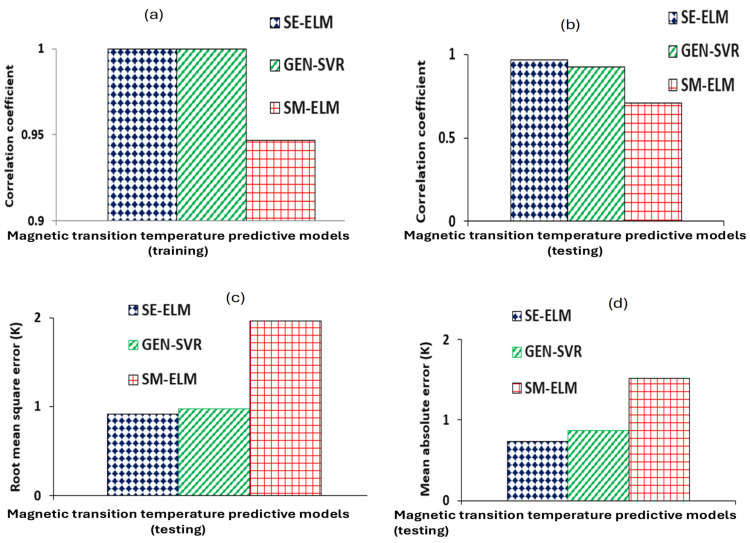
Generalization strength of the developed models (GEN-SVR, SE-ELM and SM-ELM) using (**a**) CC for training double perovskite ceramics, (**b**) CC for testing samples of double perovskite ceramics, (**c**) RMSE for testing samples of double perovskite ceramics, and (**d**) MAE for testing samples of double perovskite ceramics.

The estimation strength and predictive capacity of magnetic transition temperature predictive models for rare-earth transition metal-based double perovskite ceramics were computed using root mean square error (RMSE), correlation coefficient (CC), and mean absolute error (MAE) for training and testing samples of rare-earth transition metal-based double perovskite ceramics. The comparison is presented in Figure 5 for SE-ELM, SM-ELM and GEN-SVR predictive models. For the CC metric, shown in Figure 5a, using training rare-earth transition metal-based double perovskite ceramics, SE-ELM predictive model has an absolute CC of 100% while the GEN-SVR and SM-ELM models attained CC of 99.95% and 94.66%, respectively. Interestingly, SE-ELM predictive model further shows superior performance over other two predictive models when validated using new data samples through a test set cross-validation system of assessing generalization strength of intelligent models. As shown in Figure 5b, CC of 96.92% was obtained using the SE-ELM model, while the GEN-SVR and SM-ELM predictive models attained 92.84% and 71.16%, respectively. Based on RMSE and MAE, using testing samples of rare-earth transition metal-based double perovskite ceramics—as presented in Figure 5c and Figure 5d, respectively—the SE-ELM model attains RMSE of 0.9165 K compared to 0.9783 K for the GEN-SVR predictive model and 1.9611 K for the SM-ELM model. Similarly, the SE-ELM predictive model attains MAE of 0.7355 K, while the GEN-SVR and SM-ELM models, respectively, attained MAE of 0.8721 K and 1.5189 K.

On the basis of performance superiority, the SE-ELM magnetic transition temperature predictive model performs better than the GEN-SVR predictive model, with an improvement of 0.046% and 4.2125%, using CC metric on training and testing samples of rare-earth transition metal-based double perovskite ceramics, respectively. On the testing samples of rare-earth transition metal-based double perovskite ceramics, the SE-ELM predictive model outperforms the GEN-SVR model with an improvement of 6.3252% (using RMSE metric) and 15.6661% (using MAE metric). The SE-ELM predictive model further outperforms the SM-ELM model, with an improvement of 5.3441% using CC computed with training samples. For the testing samples of rare-earth transition metal-based double perovskite ceramics, SE-ELM outperforms the SM-ELM predictive model, with an improvement of 26.579% (using CC metric), 53.2679% (using RMSE metric), and 51.5744% (using MAE metric). Similarly, GEN-SVR predictive model outperforms the SM-ELM model with superiority of 5.3005% using CC computed with training samples of rare-earth transition metal-based double perovskite ceramics. Using testing samples of rare-earth transition metal-based double perovskite ceramics, the GEN-SVR predictive model performs better than the SM-ELM model by 23.3501% (using CC metric), 50.1124% (using RMSE metric), and 42.5786% (using MAE metric). Table 3 presents the performance measuring parameters and their associated computed metrics. The performance superiorities of each of the predictive model are also contained in the table.

### 4.3. Computed Predictions of SE-ELM, GEN-SVR, and SM-ELM Models and Comparison with the Measured Magnetic Transition Temperatures

The predictions of each model are presented in Table 4, along with the absolute difference between the measured and predicted values. The estimates of SE-ELM model agreed excellently well with the measured magnetic transition temperature, except for a few ceramics, where slight deviations were obtained.

Ceramics with little deviation from the measured magnetic transition temperature include Tm_2_FeCrO_6_ with measured and predicted magnetic transition temperature of 10.5 K [63] and 10.6 K, respectively, Ho_2_CoMnO_6_ with measured and predicted magnetic transition temperature of 8.0 K [29] and 6.8 K, respectively, Gd_2_FeCoO_6_ with measured and predicted magnetic transition temperature 4.9 K [67] and 4.3 K, respectively, Dy_2_ZnMnO_6_ with measured and predicted magnetic transition temperature 10.4 K [62] and 11.9 K, respectively, and Ho_2_NiMnO_6_ with measured and predicted magnetic transition temperature 5.5 K [19] and 5.3 K, respectively. For the SM-ELM predictive model, magnetic transition temperature of many rare-earth transition metal-based double perovskite ceramics were predicted exactly with zero deviation from the measured values. Ceramics with exactly predicted magnetic transition temperature include Ho_2_CrMnO_6_ [61], Gd_2_ZnMnO_6_ [62], Er_2_FeCrO_6_ [63], Gd_2_FeAlO_6_ [65], Ho_2_ZnMnO_6_ [62], Er_2_FeCoO_6_ [67], Er_2_CrMnO_6_ [61] and Ho_2_FeAlO_6_ [65]. The predicted magnetic transition temperature of the remaining ceramics shows slight deviation. The predicted magnetic transition temperature using GEN-SVR model agree well with the measured values especially with Ho_2_CrMnO_6_ [61] ceramic. Other investigated ceramic samples show deviation of 0.2 K except Dy_2_CuMnO_6_ [64] with 0.1 K deviation, Er_2_CrMnO_6_ [61] with 0.9 K deviation, Ho_2_ZnMnO_6_ [62] with 1.6 K deviation, Dy_2_NiMnO_6_ [19] with 0.1 K deviation and Ho_2_NiMnO_6_ [19] with 0.6 K deviation. The overall mean absolute deviation of the SE-ELM, GEN-SVR and SM-ELM predictive models are 0.1 K, 0.3 K and 0.7 K, respectively. ELM model has demonstrated superior performance over SVR-based model due to different error minimization principle adopted by the algorithms. SVR minimizes training error using structural risk principle while empirical risk principle forms the foundation of ELM-based model. Similarly, sine activation function employed by the SE-ELM model transforms to better predictive strength as compared with sigmoid activation function utilized in the SM-ELM model.

### 4.4. Dependence of Magnetic Transition Temperature on Applied Magnetic Field Using the SE-ELM Predictive Model

**Figure 6:**
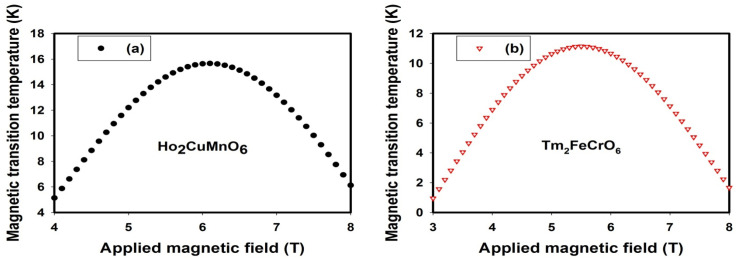
Dependence of magnetic transition temperature on magnetic field for (**a**) Ho_2_CuMnO_6_ (**b**) Tm_2_FeCrO_6_ rare-earth transition metal-based double perovskite ceramics.

Dependence of magnetic transition temperature on applied magnetic field on rare-earth transition metal-based double perovskite ceramics was investigated using developed SE-ELM predictive model. Understanding this relationship is of great significance in optimizing and tailoring rare-earth transition metal-based double perovskite ceramics for specific applications such as magnetic sensors, magnetic refrigeration, data storage, and spintronics among other cryogenic applications. Through the establishment of dependence of magnetic transition temperature and applied magnetic field, rare-earth transition metal-based double perovskite ceramic can be tailored for designing magnetic sensors that can detect changes in magnetic fields, magnetic memory devices with high stability and storage density, and spintronics devices such as tunnel junctions. Figure 6 presents the magnetic field dependence on magnetic transition temperature using the SE-ELM predictive model for Ho_2_CuMnO_6_ (shown in Figure 6a) and Tm_2_FeCrO_6_ (shown in Figure 6b).

Increase in applied magnetic field results in an increase in magnetic transition temperature due to the alignment of magnetic moment in Ho_2_CuMnO_6_ (shown in Figure 6a) and Tm_2_FeCrO_6_ (shown in Figure 6b) ceramics. Further increase in applied magnetic field results in a disruption in magnetic interaction and consequently lowers the magnetic transition temperature. The results of modeling and simulation presented in Figure 6a (for Ho_2_CuMnO_6_ ceramic) and Figure 6b (for Tm_2_FeCrO_6_ ceramic) correspond with the reported experimental values at 5T [63,64]. This investigation employs the weights generated during pattern acquisition in SE-ELM predictive models. Similar observation can be investigated using different samples of rare-earth transition metal-based double perovskite ceramics. The weights associated with the SE-ELM and SM-ELM predictive models are contained in the Appendix A in case of further investigation with newly synthesized or existing on rare-earth transition metal-based double perovskite ceramics.

## 5. Conclusions

Magnetic transition temperature of E_2_TMO_6_ rare-earth transition metal-based double perovskite ceramics is modeled through genetically hybridized support vector regression (GEN-SVR) and extreme learning machine (ELM) intelligent algorithms. The developed predictive models include sine activation function-based ELM (SE-ELM), sigmoid activation function-based ELM (SM-ELM) and GEN-SVR models. The performance of the predictive models was evaluated and assessed using root mean square error (RMSE), correlation coefficient (CC), and mean absolute error (MAE) performance metrics. For the testing samples of double perovskite ceramics, SE-ELM outperforms the SM-ELM predictive model, with an improvement of 26.6% (using CC metric), 53.3% (using RMSE metric), and 51.6% (using MAE metric). SE-ELM predictive model outperforms GEN-SVR model with superiority of 6.3% (using RMSE metric) and 15.7% (using MAE metric). Similarly, the GEN-SVR predictive model performs better than the SM-ELM model with enhancement of 23.4% (using CC metric), 50.1% (using RMSE metric), and 42.6% (using MAE metric). The influence of magnetic field on magnetic transition temperature was investigated for Ho_2_CuMnO_6_ and Tm_2_FeCrO_6_ ceramics. The developed predictive models are only limited to magnetic transition temperature prediction of E_2_TMO_6_ rare-earth transition metal-based double perovskite ceramics. The demonstrated precision of the developed predictive models coupled with the established magnetic field dependence on magnetic transition temperature is crucial in designing E_2_TMO_6_ double perovskite ceramics for several cryogenic applications such as magnetic sensors, data storage, spintronics and magnetic refrigeration for addressing global energy crisis.

## Figures and Tables

**Table 1:** Data sample analysis for all input descriptors and measured magnetic transition temperature.

	Magnetic Transition Temperature (K)	H (T)	E	T	M
Mean	6.47	5.96	104.67	77.76	77.91
Maximum	12.20	7.00	107.80	94.00	94.00
Standard deviation	2.86	1.02	1.75	7.17	5.83
Minimum	2.00	5.00	102.00	68.00	67.50
Correlation coefficient	1.00	−0.39	−0.14	−0.12	0.60

**Table 2:** Global solutions obtained from genetic algorithm optimization for GEN-SVR model.

Hyperparameter	Optimum Value
Penalty factor	7970.066
Chromosome size	100
Epsilon	0.154694
Option	1
Function	Gaussian
hyper-plane	E-7

**Table 3:** Generalization strength comparison between the developed magnetic transition temperature predictive models.

	Training	Testing Testing Testing		
	CC	CC	RMSE	MAE
SE-ELM	100.00	96.92	0.92	0.74
GEN-SVR	99.95	92.84	0.98	0.87
SM-ELM	94.66	71.16	1.96	1.52
Superiority of SE-ELM over GEN-SVR	0.05	4.21	6.33	15.67
Superiority of SE-ELM over SM-ELM	5.34	26.58	53.27	51.57
Superiority of GEN-SVR over SM-ELM	5.30	23.35	50.11	42.58

**Table 4:** Predicted magnetic transition temperature (MTT) of rare-earth transition metal-based double perovskite ceramics and their comparison with the measured values.

Compound	MTT (K)	SE-ELM	SE-ELM Residual	SM-ELM	SM-ELM Residual	GEN-SVR	GEN-SVR Residual
Ho_2_CrMnO_6_	6.1 [61]	6.1	0.0	6.1	0.0	6.1	0.0
Gd_2_ZnMnO_6_	6.4 [62]	6.4	0.0	6.4	0.0	6.2	0.2
Er_2_FeCrO_6_	11.7 [63]	11.7	0.0	11.7	0.0	11.5	0.2
Er_2_CuMnO_6_	3.6 [64]	3.6	0.0	5.3	1.7	3.8	0.2
Er_2_CoMnO_6_	7.5 [29]	7.5	0.0	6.5	1.0	7.3	0.2
Ho_2_NiMnO_6_	6.0 [19]	6.0	0.0	5.5	0.5	5.4	0.6
Dy_2_FeAlO_6_	7.8 [65]	7.8	0.0	7.8	0.0	7.6	0.2
Dy_2_NiMnO_6_	6.0 [19]	6.0	0.0	5.6	0.4	5.9	0.1
Dy_2_CoMnO_6_	5.0 [29]	5.0	0.0	5.4	0.4	5.2	0.2
Gd_2_FeAlO_6_	2.0 [65]	2.0	0.0	2.0	0.0	2.2	0.2
Ho_2_ZnMnO_6_	6.8 [62]	6.8	0.0	6.8	0.0	8.4	1.6
Er_2_NiMnO_6_	5.0 [19]	5.0	0.0	6.3	1.3	5.2	0.2
Gd_2_CuMnO_6_	7.5 [64]	7.5	0.0	7.4	0.1	7.3	0.2
Er_2_NiMnO_6_	5.0 [19]	5.0	0.0	5.7	0.7	5.2	0.2
Ho_2_FeCoO_6_	4.0 [66]	4.0	0.0	3.9	0.1	4.2	0.2
Er_2_FeCoO_6_	2.7 [67]	2.7	0.0	2.7	0.0	2.9	0.2
Er_2_CrMnO_6_	5.2 [61]	5.2	0.0	5.2	0.0	6.1	0.9
Ho_2_FeAlO_6_	2.0 [65]	2.0	0.0	2.0	0.0	2.2	0.2
Dy_2_CuMnO_6_	12.1 [64]	12.1	0.0	13.5	1.4	11.1	1.0
Dy_2_NiMnO_6_	6.0 [19]	6.0	0.0	5.5	0.5	6.2	0.2
Gd_2_FeCoO_6_	4.9 [67]	4.9	0.0	5.0	0.1	5.1	0.2
Ho_2_CuMnO_6_	12.2 [64]	12.2	0.0	9.2	3.0	12.0	0.2
Tm_2_FeCrO_6_	10.5 [63]	10.6	0.1	12.2	1.7	10.3	0.2
Ho_2_CoMnO_6_	8.0 [29]	6.8	1.2	6.4	1.6	7.8	0.2
Gd_2_FeCoO_6_	4.9 [67]	4.3	0.6	5.5	0.6	5.1	0.2
Dy_2_ZnMnO_6_	10.4 [62]	11.9	1.5	6.7	3.7	10.2	0.2
Ho_2_NiMnO_6_	5.5 [19]	5.3	0.2	5.4	0.1	5.7	0.2
**Mean absolute error (K)**			**0.1**		**0.7**		**0.3**

## Data Availability

The original contributions presented in this study are included in the article. Further inquiries can be directed to the corresponding author.

## Appendix A

**Table A1:** SE-ELM predictive model.

k	Biases	Output Weight	Magnetic Field Weight	Rare-Earth Weight	Transition Metal Weight	Any Metal Weight
1	0.7225130617	1.2427827374	0.1148810873	−0.3055849693	−0.4482516227	−0.3552237815
2	0.6849300618	−1.2434195665	0.5617067582	0.9311123273	−0.6659882046	−0.3743215913
3	0.1373017009	−0.9468194910	−0.7209497397	0.6701749553	0.1015746494	0.5451241719
4	0.3599920346	−0.8540009678	0.9754589655	0.6552374364	0.6345787698	0.7214028533
5	0.7907223920	−1.7162123937	0.5423318793	0.3483318196	−0.6136811884	0.9418167148
6	0.5750831796	−0.1828785703	−0.0829940562	−0.9131051465	−0.9170666451	−0.1276684793
7	0.7662664029	−0.1886235946	−0.3753636682	0.5743482016	0.5065171562	0.7923093773
8	0.3746464268	−2.0139427494	0.3628797001	0.8929218978	0.9170336391	0.9389560284
9	0.3788092433	1.5966158208	0.5453768478	−0.9265712958	0.6234800595	0.5056407731
10	0.2536340812	−2.4636479977	−0.5706482370	−0.4988611117	0.0145844402	−0.2040967660
11	0.0851201958	−1.9694387409	0.2371332802	−0.8610782845	0.7489166993	−0.4903197092
12	0.5709101967	0.9993483906	−0.0777118592	0.2285536523	0.3535786575	−0.2811934590
13	0.0961277755	−0.8102393115	0.6526073931	−0.5661174172	−0.0803850368	−0.2983802274
14	0.9619358872	0.0098323448	0.4677085810	0.6354738468	−0.1657509879	−0.9582320391
15	0.4707914879	−0.9885653735	0.9540538818	0.6839049860	0.8879276198	0.7799287663
16	0.9907834343	0.1539170883	−0.7996574998	0.8301807255	−0.7243864790	0.2819266159
17	0.6678873888	−1.1401651798	−0.3234151686	−0.8743317604	0.6950044196	−0.3578771669
18	0.2376634038	−2.5425554676	−0.2767898283	−0.9726729576	0.7707882551	−0.6746939395
19	0.8323018721	1.3888443024	−0.4830145061	0.3207876845	0.2593221792	0.4063154274
20	0.8194160401	−0.3749088644	0.0116857832	0.0526458289	0.9834831412	−0.4806278364
21	0.0971327487	2.2993310034	−0.7055143547	−0.5006812624	−0.0324958504	−0.0889978758
22	0.7127082668	−0.6617365338	−0.1490523076	−0.7719032061	−0.3058343469	0.9576661155
23	0.1340897564	1.5865820472	0.2140103342	−0.1862000203	−0.6480876838	0.1971435022
24	0.0908430111	0.1061975617	0.2004428755	0.5649868639	0.1986894682	0.8913778924
25	0.1763236663	1.4488020633	0.1456248639	0.6091153115	−0.9922893515	0.7853739345
26	0.3369806420	0.0170290950	−0.9517564978	−0.6518093938	0.7986093796	0.0718938964
27	0.3993371707	−1.1988152483	0.1083380068	0.3303917366	0.9758626664	0.1973593519
28	0.8156553780	−1.2920576406	−0.9893664948	0.4878220974	0.5938554601	−0.0111496625
29	0.8616999802	0.8958653101	−0.8782046097	−0.8199701610	0.7457298903	−0.3736714070
30	0.2959886774	1.8906007808	−0.7107386440	−0.6714505524	0.5855811204	0.2868872656
31	0.8027759212	−0.3547897695	−0.1767079645	−0.0553678761	0.0374571552	−0.6203722170
32	0.4577495535	−1.2054657890	−0.1451153480	0.2015119160	−0.8250790407	0.1787523728
33	0.5550028032	−0.6662053968	−0.9912670099	−0.6222089491	0.1131391622	−0.4404763277
34	0.6299283784	−0.8174409202	−0.3841335379	0.8541546066	−0.3748884618	0.4725273257
35	0.5235554725	0.7602517861	−0.3363700556	0.2798180761	−0.7516126422	0.8595361434
36	0.0424642718	1.6497831412	−0.7371612765	−0.5793845617	0.8957253281	−0.3219466027
37	0.0915139834	0.3941299563	0.9751241704	−0.8004130391	−0.8693519293	−0.0690114476
38	0.4281027425	0.1463530322	0.5421960181	0.9561890553	0.8029879394	0.1257953417
39	0.5275663121	−1.0685898382	−0.4911547089	0.9272725271	0.8448522109	0.0587709823
40	0.1603795738	0.9283264592	0.1849563719	−0.3475082642	0.3965291135	0.3069042295
41	0.4305470222	0.4131597378	−0.5995995543	0.7244229740	−0.8321645532	0.9202493589
42	0.2068522910	0.2696125287	−0.3370570009	−0.4464422039	−0.2413942285	−0.4854087816
43	0.4290686318	1.1591516256	0.7986277631	0.3216888986	0.2778271091	−0.4802365031
44	0.4770785345	−0.9223261932	0.6241152260	−0.7863318218	−0.5401958250	0.3819927897
45	0.4159877211	−0.5150763561	0.6444774872	0.2565413758	0.6034543746	0.0052674018
46	0.1784944762	−0.8020380120	−0.2233216232	0.1447972871	0.8271586672	−0.6615341834
47	0.1457612105	0.3408460247	0.0216856667	−0.5782159655	−0.6303544444	−0.6247065050
48	0.4023157185	1.6104171411	−0.7846329653	−0.4301464485	0.5429205965	−0.5299353098
49	0.6686333493	0.6502204882	0.7275401732	−0.1850086859	−0.5542099123	0.5078068078
50	0.0332940348	−0.1509337001	0.4192856973	0.6203744041	−0.9066815615	0.6009994086
51	0.5225941328	1.1298387384	0.6785805165	0.5402834724	−0.4368491298	−0.3836899471
52	0.3953559593	0.5474267870	−0.8001374564	0.4103617836	0.9035865611	0.1532144541
53	0.7039555868	0.4711073221	−0.4430033100	0.8014009430	0.8652038311	−0.5053491936

**Table A2:** SM-ELM predictive models.

k	Biases	Output Weight	Magnetic Field Weight	Rare-Earth Weight	Transition Metal Weight	Any Metal Weight
1	0.495103	28.212456	−0.479931	0.101244	−0.964970	0.929804
2	0.920545	−10,466,701.398800	−0.995693	−0.802332	−0.937378	0.796610
3	0.550515	−545,220.272782	0.414773	−0.483924	−0.059765	−0.484183
4	0.328303	−1,933,091,513.243830	0.992638	0.115491	0.983123	−0.207775
5	0.851456	−1,930,103,987.452320	−0.472958	0.753577	−0.760417	0.475394
6	0.489914	−1,929,438,129.932470	0.641215	0.631100	−0.459157	0.917257
7	0.193632	−1,929,820,803.582070	−0.191521	0.532867	0.518408	0.609534
8	0.509486	10,591.377512	0.981898	−0.650621	0.070397	−0.790181
9	0.482300	−41,322,937,065.435100	−0.933548	−0.107854	0.199715	−0.307776
10	0.927984	−5,547,088,752.814350	0.539871	0.902726	−0.790297	−0.841089
11	0.923499	6.321526	−0.499757	−0.480659	−0.217418	−0.734662
12	0.236260	11,588.533673	−0.288171	−0.041518	0.221237	−0.024587
13	0.516165	11,397,360.963912	0.517438	0.783815	−0.822271	−0.810507
14	0.862271	−1,924,164,276.895450	−0.438467	0.981014	−0.141672	−0.635907
15	0.903994	−697,562.494739	0.243786	−0.856419	0.403565	0.977871
16	0.071308	−1,929,801,307.588630	0.522076	0.620839	−0.272625	0.421814
17	0.498830	−0.000016	0.404737	−0.443972	−0.184315	−0.419679
18	0.954388	−1,929,801,307.589110	0.219814	0.590575	0.496875	0.701237
19	0.262015	−1,929,801,307.589120	−0.852440	0.846436	−0.187352	0.430702
20	0.881432	−1,408,516.015777	−0.456174	0.606453	−0.663270	0.102670
21	0.990681	−1,929,801,307.589120	−0.334289	0.504171	0.138129	0.352268
22	0.003085	−1,929,801,307.589120	−0.222435	0.446963	0.927462	0.161070
23	0.546374	−1,929,801,307.589120	0.575198	0.573692	0.760851	−0.637897
24	0.213634	−1,929,801,307.589120	−0.563331	−0.285640	0.359468	0.799460
25	0.174239	0.000000	−0.046091	−0.827885	−0.630638	0.562965
26	0.641141	−21,629,467.263229	0.053857	−0.277972	0.988591	−0.321922
27	0.119745	−35,206,996.791366	0.787812	−0.627138	0.788610	−0.526778
28	0.199852	−50.706935	0.505250	0.049929	−0.671512	0.644013
29	0.410902	1.502535	−0.944841	−0.306882	−0.806581	0.548474
30	0.447623	0.819332	0.631349	0.366138	−0.584833	−0.657705
31	0.689286	0.000000	0.405988	−0.856346	0.352394	−0.250506
32	0.772646	−895,325,993.235744	0.053254	0.007612	−0.338845	0.799921
33	0.209038	−1,929,801,307.589120	0.914569	0.790592	−0.339547	0.188207
34	0.595910	−1,929,801,307.589120	−0.875421	0.703366	−0.195368	−0.008984
35	0.738968	−0.000009	−0.991784	−0.960065	0.925270	−0.468429
36	0.590238	0.000000	0.520370	−0.603955	−0.341003	−0.313557
37	0.950994	−0.301471	0.927915	−0.438877	−0.296218	0.817231
38	0.108907	7,832,794.611025	0.643694	0.347742	0.006215	−0.802348
39	0.967554	−1,929,801,307.589120	0.062504	0.991729	0.960567	0.426048
40	0.528661	−1,929,801,307.589120	−0.827708	−0.447798	0.637542	0.932061
41	0.034482	45,519,425,253.511100	0.392963	0.921239	−0.814654	−0.048659
42	0.066202	−149,341.524572	−0.304685	−0.002606	−0.273450	0.136545
43	0.041879	0.000000	0.371048	0.190920	−0.762302	−0.430662
44	0.302667	−12.302782	0.560988	0.232797	0.677680	−0.872136
45	0.340062	−89,457,091.390429	0.492860	0.795073	−0.921520	0.157988
46	0.408860	−1,929,801,307.589120	0.431660	0.165800	0.587793	0.419660
47	0.038721	−7,847,081,357.644930	0.215880	−0.741172	0.871389	0.568222
48	0.883392	−1,929,801,307.589120	0.609993	0.432708	0.726688	0.079436
49	0.227191	−55,056,957.589018	0.039576	−0.540297	0.901206	−0.499696
50	0.621551	208.011219	−0.668210	−0.060474	0.980425	−0.677664
51	0.702999	−277,631,017,251.814000	0.194818	0.113756	0.268420	−0.863770
52	0.981236	−1,929,801,307.589120	0.651614	0.092626	0.152455	0.683079
